# Cellular therapies for bone repair: current insights

**DOI:** 10.1186/s10195-024-00768-0

**Published:** 2024-05-24

**Authors:** Paul Rodham, Farihah Khaliq, Vasileos Giannoudis, Peter V. Giannoudis

**Affiliations:** 1 Academic Department of Trauma and Orthopaedics, School of Medicine, University of Leeds, Leeds, UK; 2 Academic Department of Trauma and Orthopaedic Surgery, School of Medicine, University of Leeds, Leeds, UK; 3 NIHR Leeds Biomedical Research Centre, Chapel Allerton Hospital, Leeds, UK

**Keywords:** Stem cells, Cellular therapy, Fracture healing, Clinical applications

## Abstract

Mesenchymal stem cells are core to bone homeostasis and repair. They both provide the progenitor cells from which bone cells are formed and regulate the local cytokine environment to create a pro-osteogenic environment. Dysregulation of these cells is often seen in orthopaedic pathology and can be manipulated by the physician treating the patient. This narrative review aims to describe the common applications of cell therapies to bone healing whilst also suggesting the future direction of these techniques.

## Introduction

The presence of a subset of non-haematopoietic stem cells within the bone marrow was a concept first suggested in 1867 by the German pathologist Friedrich Conheim [1]. However, it was not until 1970 that Alexander Friedenstein found that this population of cells demonstrate plastic adherence, dividing and forming small colonies in culture [2]. “Mesenchymal stem cell” was a term first coined by Arnold Caplan in 1991, who demonstrated the ability of these cells to undergo trilineage differentiation into osteoblasts, adipocytes and chondrocytes [3]. More recently, however, it has been established that this cell population is heterogeneous, possessing only a few true stem cells, which cannot be differentiated as yet; therefore, the term “mesenchymal stromal cell” (MSC) is preferred [4]. In 2006, the International Society for Cellular Therapy (ISCT) defined MSCs as cells with the following characteristics [5]:

1. They adhere to plastic in standard culture
2.  ≥ 95% of the cell population express CD73, CD90 and CD105, whereas < 2% express CD14, CD19, CD34, CD45 and HLA-DR
3. They are capable of in vitro differentiation into osteoblasts, chondroblasts and adipocytes under standard differentiating conditions.

Lately, a lot of attention has been given to the role of MSCs in bone repair. Bone is a unique tissue within the human body that can heal and regenerate without forming scar tissue. Key to this healing response is the recruitment of both local and remote MSCs to the site of injury, where they can differentiate into osteoblasts and produce local pro-osteogenic trophic factors [6]. Chemotaxis of MSCs is mediated by the SDF-1/CXCR4 signalling pathway, and the CXCR4 expression by MSCs is associated with improved homing capacity [7]. Interestingly, this receptor is downregulated in culture-expanded MSCs, which have been demonstrated to have poorer homing abilities [8]. MSCs share several signalling pathways with immune cells, ensuring that they are recruited alongside these immune cells during the inflammatory phase. One of the more important is monocyte chemoattractant protein-1, which allows binding to CCR2 on the vascular endothelium, where MSCs can translocate into the target tissue [9]. Pro-inflammatory cytokines, including interferon-γ and TNF-α, increase the production of matrix metalloproteinase (MMP), which allow MSCs to migrate through the extracellular matrix (ECM) [10]. In a pro-inflammatory environment, MSCs produce numerous immunomodulatory substances, including prostaglandin E2, indoleamine 2,3-dioxygenase, nitric oxide and TGF-β [11]. Through their direct differentiation into bone progenitor cells and modulation of the local inflammatory cytokine environment, MSCs are critical to bone healing and repair.

MSCs, however, are not the sole contributors to bone healing. Used as an injectable, MSCs can provide an osteogenic stimulus to a healing bone; however, as per the diamond concept of bone healing, MSCs must exist in an environment that provides sufficient osteoinductive signals and must be provided with an osteoconductive scaffold that can encourage ingrowth, appropriate mechanical stability and a well-vascularised bed [12]. As such, MSCs can be combined with a number of substances to optimise their capabilities in bone repair. Autograft, in either its cortical or cancellous form, provides a source of osteogenic cells, although the number of them is reduced during harvest and transfer. Autograft provides the perfect osteoconductive scaffold onto which MSCs can be seeded to circumvent this problem and demonstrates osteoinductive properties that encourage MSC proliferation and differentiation [13]. Similarly, allograft can be utilised as a scaffold—either as an unprocessed graft or in a more processed form such as demineralised bone matrix (DBM)—to provide osteoconductivity, albeit with limited osteoinductivity [14]. Further osteoinductivity can be achieved through the combination of bone morphogenetic protein (BMP) or platelet-rich plasma (PRP) with cells and allograft [15, 16].

The use of both autograft and allograft is limited due to limited graft availability or immunogenicity; therefore, more recently, there has been an increase in the use of synthetic carriers. These scaffolds aim to mimic the structure of the local extracellular matrix (ECM), providing an osteoconductive structure onto which MSCs migrate and proliferate. Through their surface topography, scaffolds can influence the differentiation of MSCs via mechanotransduction, directing MSC differentiation towards an osteoblastic lineage [17]. At present, scaffolds commonly used in the delivery of MSCs include bioceramics (commonly hydroxyapatite or β-tricalcium phosphate (β-TCP)), biodegradable polymers (such as polylactic acid (PLA) and polycaprolactone (PCL)), and composite biomaterials (combinations of ceramics and polymers) [18]. Bioprinting three-dimensional computer-aided design (CAD) scaffolds with impregnated MSCs represents an exciting frontier, but, as of yet, it is not established in routine practice [19].

**Fig. 1:**
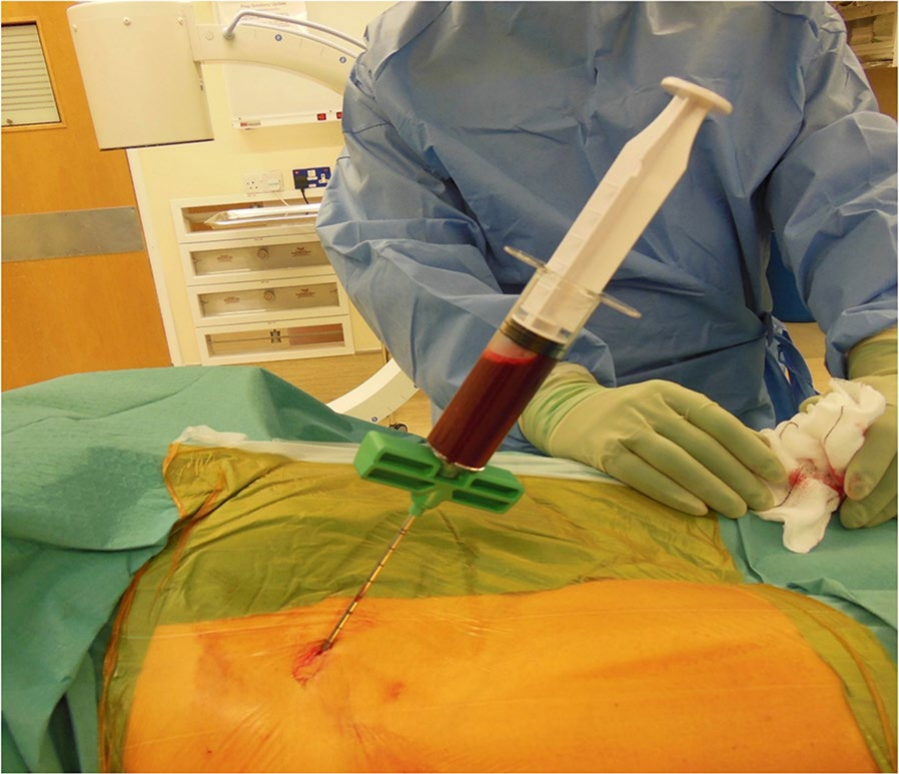
Intraoperative image showing aspiration of bone marrow aspirate from the anterior pelvic iliac crest

MSCs can be harvested from several sites, of which the iliac crest is the most commonly utilised due to its ease of access (Fig. 1). Once harvested, the cells can be either injected directly or expanded ex vivo and reimplanted at a later date [20].

In our institution, we use the anterior and/or the posterior iliac crest of the pelvis as the harvesting site of the MSCs. The patient is placed in either the lateral or supine position. A stylet with its trocar point is inserted into the iliac crest and the bone marrow is aspirated into two 30-ml syringes. Prior to aspiration, each 30-ml syringe is prefilled with 6 cc of ACD-A for a total of 60 ml of anticoagulated marrow. The aspirate volume is then transferred to a tube, and the MarrowStim concentration system (Zimmer Biomet, Warsaw, IN, USA) is used for centrifugation. After a spinning time of 15 min, a volume of 7 ml of concentrated marrow containing MSCs is extracted from the tube [21]. The concentrated bone marrow can then be either directly injected into the site of interest or loaded onto a scaffold for delivery to the site of nonunion. Our previous work enumerating CD45^low^CD271^high^ cells using the Attune-based method showed a median of 1520 cells/ml of bone marrow (95% CI: 1056 to 6112; range: 96 to 20,992 cells/ml of bone marrow) [22].

Direct injection has the advantage of requiring only a single-stage procedure, but there are concerns regarding the containment of the cells with this technique, particularly when aiming to address bone defects where a scaffold would be beneficial in guiding cell localisation. Ex-vivo expansion of MSCs allows for greater cell yields that can be loaded onto scaffolds for the management of bone defects. The cells can adhere to the scaffold, providing containment and often encouraging their osteogenic differentiation. There are, however, concerns that cells lose their potency with increased time in tissue culture, with many opting to perform the second stage after one or two passages [13].

The aim of this narrative review is to describe the current applications of cell therapy for bone repair, examining current practice in harvest, application and supplementary therapy as well as clinical results.

## Materials and methods

This scoping review was conducted in accordance with the guidance described in the* Cochrane Handbook of Systematic Reviews*. A search of the relevant electronic databases (Ovid, Medline and PubMed) was conducted using keywords relating to MSC or marrow aspirate in bone-healing applications. Articles were identified through the screening of titles and abstracts, with full texts retrieved for those articles relevant to this study.

Inclusion criteria included all studies published in the English language since 2010 that assessed the use of progenitor cells or marrow aspirate for the augmentation of bone healing. Once identified, data were extracted, including the condition treated, the product used (culture-expanded cells vs marrow aspirate), supplementary therapy, duration of follow-up, and both radiological and clinical outcomes. These data are summarised in Tables 1–10.

**Table 1:** Papers assessing the use of cell therapies in acute fracture care

Authors	Procedure	Sites treated	Source of MSCs	Supplementary therapy
Shim et al. 2021 [24]	Teriparatide ± MSC intramedullary + intravenous injection for osteoporotic vertebral fractures	Experimental—7 Control—7	Wharton-jelly-derived MSCs harvested at passage 7	Both control and experimental groups received teriparatide
Verma et al. 2017 [28]	Bone marrow injection during fixation of the intracapsular neck of femur fractures	Experimental—16 Control—16	Anterior iliac crest, direct injection	All fractures fixed with cannulated screws, no other supplementary therapy
Seebach et al. 2016 [29]	MSCs in plate-stabilised proximal humeral fractures	Proximal humerus—10	Posterior iliac crest, ex-vivo expanded, sited on β-TCP scaffold	No other supplementary therapy
Libergall et al. 2013 [25]	Injection of MSCs into acute tibial fractures at 3–6 weeks post-IMN	Experimental—12 Control—12	Anterior iliac crest—MSCs were flow sorted to ensure purity	Experimental—DMB and PRP Control—no intervention at the 3- to 6-week mark
Kim et al. 2009 [26]	Injection of autologous cultured osteoblasts into long-bone fractures 6–8 weeks post-fixation	Experimental: Tibia—13 Femur—11 Ulna—4 Humerus—2 Radius—1 Control: Tibia—18 Femur—9 Radius—2 Ulna—2 Humerus—1 Fibula—1	Anterior iliac crest, culture expanded and harvested at P1	No supplementary therapy to experimental group except for injection of cells Control group underwent standard care

*IMN* Intramedullary Nailing

### MSCs in acute fracture healing

Given their key role in osteogenic differentiation and the control of the local paracrine environment, direct implantation of MSCs into acute fractures has been an area of particular interest (Tables 1 and 2). Osteoporotic vertebral fractures are endemic and can lead to significant pain and disability [23]. To try and improve outcomes in this cohort, Shim et al. performed both local and systemic injection of Wharton-jelly-derived MSCs in combination with systemic administration of the synthetic parathyroid hormone (PTH) teriparatide [24]. Compared to teriparatide therapy alone, the experimental group demonstrated significantly improved pain scores, Oswestry disability index scores, and bone microarchitecture on CT at both 6 and 12 months. A number of authors suggest injecting MSCs in the early period following fracture fixation in an attempt to accelerate bone healing and facilitate a return to normal function. Liebergall et al. injected flow-selected MSCs into acute tibial fractures at between 3 and 6 weeks post-operatively and noted a reduction in the time to union of nearly half [25]. Similarly, Kim et al. examined the injection of cultured osteoblastic cells into a number of long-bone fractures (mostly tibias and femurs) at 6 to 8 weeks following the index procedure and also noted a significantly faster rate of radiological healing compared to standard treatment [26].

**Table 2:** Outcomes following the use of cell therapies for acute fracture care

Authors	Duration of follow-up	Radiological outcome	Clinical outcome
Shim et al. 2021 [24]	12 months	Improved microarchitecture in experimental group on CT at 6 and 12 months No difference in improvement in hip and lumbar spine DEXA	Significantly greater improvement in pain scores in the experimental group Significantly greater improvement in the Oswestry disability index in the experimental group
Verma et al. 2017 [28]	19.6 months	Nonunion: 4 × nonunion in each group 1 × AVN in each group	No difference in Harris hip score between groups at the final follow-up
Seebach et al. 2016 [29]	12 weeks	All fractures healed by 12 weeks	Average DASH score by 12 weeks: 52
Libergall et al. 2013 [25]	12 months	All fractures healed by 12 months Experimental group—2.2 months to union Control group—4 months to union	No difference in pain or SF-12 scores
Kim et al. 2009 [26]	Not stated	Callus formation score used Statistically faster rate of healing in the experimental group	No differences in rates of complications

*DEXA* dual x-ray absorptiometry

Contrastingly, not all acute fractures appear to be associated with such positive results. Due to a retrograde blood supply, femoral neck fractures are associated with high rates of both nonunion and avascular necrosis (AVN) [27]. Verma et al. therefore attempted to improve outcomes through the application of bone marrow aspirate to the hip in young patients undergoing cannulated screw fixation of a displaced femoral neck fracture [28]. They demonstrated no difference in the rate of nonunion, AVN or Harris hip score at final follow-up, though it is worth noting that this injection was into the hip joint, with no way of discerning exactly where the cells would seed. Similarly, Seebach et al. examined the use of culture-expanded MSCs on β-TCP scaffolds following locking-plate fixation of proximal humeral fractures [29]. Whilst they were able to achieve healing in all patients by 12 weeks, functional outcomes as measured by the disabilities of the arm, shoulder and hand (DASH) score were poor when compared to comparable series [30].

### MSCs in fracture nonunion

Fracture nonunion represents a complex problem, the key to which is disruption to the bone’s normal healing mechanisms. Definitions vary, with the Food and Drug Administration (FDA) defining a nonunion as a fracture that has not gone on to heal 9 months following injury, with no radiological evidence of progression of healing for 3 consecutive months [31]. A more pragmatic definition is that of a fracture that has not gone on to heal within the usual timeframe of the injury and, in the opinion of the treating clinician, will not go on to heal without further intervention [32]. Fracture nonunion occurs due to complex interplay between biology and mechanics. Historically, it was felt that atrophic nonunion occurs due to inadequate biology and hypertrophic nonunion due to inappropriate mechanics; however, increasingly, it is becoming evident that this is not the case, with each nonunion requiring careful assessment to ascertain which aspect of the diamond concept is not being supported [33]. Where surgeons aim to augment the biology, autologous bone graft remains the gold standard as a source of osteoconductivity, osteoinductivity and osteogenic cells. MSCs are, however, lost in the process of harvesting and preparing autograft, and, as such, augmentation with MSCs can be utilised to optimise the osteogenic stimulus (Tables 3 and 4) [15].

**Table 3:** Papers assessing the use of cell therapy in nonunion

Authors	Procedure	Sites treated	Average time to nonunion surgery	Source of MSCs	Supplementary therapy
Jayankura et al. 2021 [42]	Percutaneous injection of allogenic MSCs into long-bone delayed union	Tibia—8 Humerus—5 Femur—3 Ulna—3 Fibula—2 Radius—1	6.6 months	Allogenic MSCs (ALLOB, Bone Therapeutics)	Nil Original fixation retained
Gomez-Barrena et al. 2020 [41]	Application of scaffold laden with MSCs to long-bone nonunion	Tibia—13 Femur—11 Humerus—4	14.7 months	Posterior iliac crest; culture expanded and applied at passage 2	Scaffold consisting of 20% hydroxyapatite, 80% β-TCP
Emadedin et al. 2017 [37]	Percutaneous injection of MSCs into long-bone nonunion	Femur—3 Tibia—2	24 months	Anterior iliac crest; culture expanded and applied at passage 1/2	Nil Original fixation retained
Wittig et al. 2016 [38]	Application of MSCs in a collagen scaffold to long-bone nonunion	Tibia—2 Femur—1	20 months	Posterior iliac crest; culture expanded + seeded into collagen microspheres	Platelet-rich plasma + collagen membrane to contain microspheres
Ismail et al. 2016 [40]	MSCs on hydroxyapatite granules vs autologous bone graft in atrophic nonunion	Both experimental and control groups contained: Femur—3 Tibia—1 Humerus—1	Experimental—37 months Control—10 months	Posterior iliac crest; culture expanded (passage 1: 4, passage 2: 1) + placed onto hydroxyapatite granules prior to implantation	All patients underwent revision plate fixation Control group had autologous iliac crest bone graft only
Hau et al. 2015 [36]	Marrow aspirate on freeze-dried allograft chips vs autologous bone graft for long-bone nonunion	Experimental: Femur—9 Tibia—6 Ulna—2 Humerus—1 Control: Femur—1 Tibia—5 Ulna—2 Humerus—1	Experimental—12 months Control—5 months	Posterior iliac crest; non-expanded and combined with freeze-dried allograft granules	Fixation revised in all cases to optimise stability Control group had autologous iliac crest graft
Giannotti et al. 2013 [39]	MSCs in a fibrin clot scaffold for the management of failed upper-limb nonunion procedures	Forearm—5 Humerus—3	Not stated	Anterior iliac crest; culture expanded and applied to a fibrin clot scaffold at passage 1	Augmented with autologous bone graft in 4 cases, allograft in 3 cases, and autologous graft with synthetic bone substitute in 1 case
Singh et al. 2013 [35]	Percutaneous marrow aspirate injection into long-bone nonunion	Ulna—6 Femur—3 Humerus—2 Metacarpal—1	9 months	Anterior iliac crest; directly injected into nonunion site	Nil Original fixation retained

**Table 4:** Outcomes following the use of cell therapies for nonunion

Authors	Duration of follow-up	Radiological outcome	Clinical outcome
Jayankura et al. 2021 [42]	6 months	Union achieved in 20/22 patients Improvements in tomographic union score and modified radiographic union score at 3 and 6 months	No treatment-mediated immune reactions observed, though the proportion of patients demonstrating donor-specific anti-HLA antibodies rose from 36 to 59% Two patients required further operative intervention to achieve union
Gomez-Barrena et al. 2020 [41]	12 months	Radiological healing: 7/28 at 3 months 19/28 at 6 months 26/28 at 12 months	VAS score: < 30/100 in 85.7% at 3 months < 30/100 in 89% at 6 months Average score of 6.6 at 12 months
Emadedin et al. 2017 [37]	12 months	Radiological union in 3/5 cases at an average of 8 months	No adverse advents related to implantation seen
Wittig et al. 2016 [38]	36 months	Union achieved in all cases within 12 months	All patients returned to normal function
Ismail et al. 2016 [40]	12 months	Union achieved in all cases Time to union: Experimental group—8 months Control group—11 months	Experimental group demonstrated greater functional improvements during the early post-op period (first 4 months)
Hau et al. 2015 [36]	24 months	Experimental: union achieved in 17/18 cases at an average of 3.3 months Control: union achieved in 8/9 cases at an average of 4.6 months	Two deep infections requiring debridement and suppression in experimental group One nonunion in each group requiring revision
Giannotti et al. 2013 [39]	76 months	8/9 patients progressed to union without further intervention	One patient required revision of radius nonunion at 6 months, having previously been managed for nonunion of their radius and ulna No late refractures seen in long-term follow-up
Singh et al. 2013 [35]	Not reported	Union achieved in 10/12 cases at an average of 4 months	2 × failures, one of which underwent revision fixation + bone grafting

**Fig. 2:**
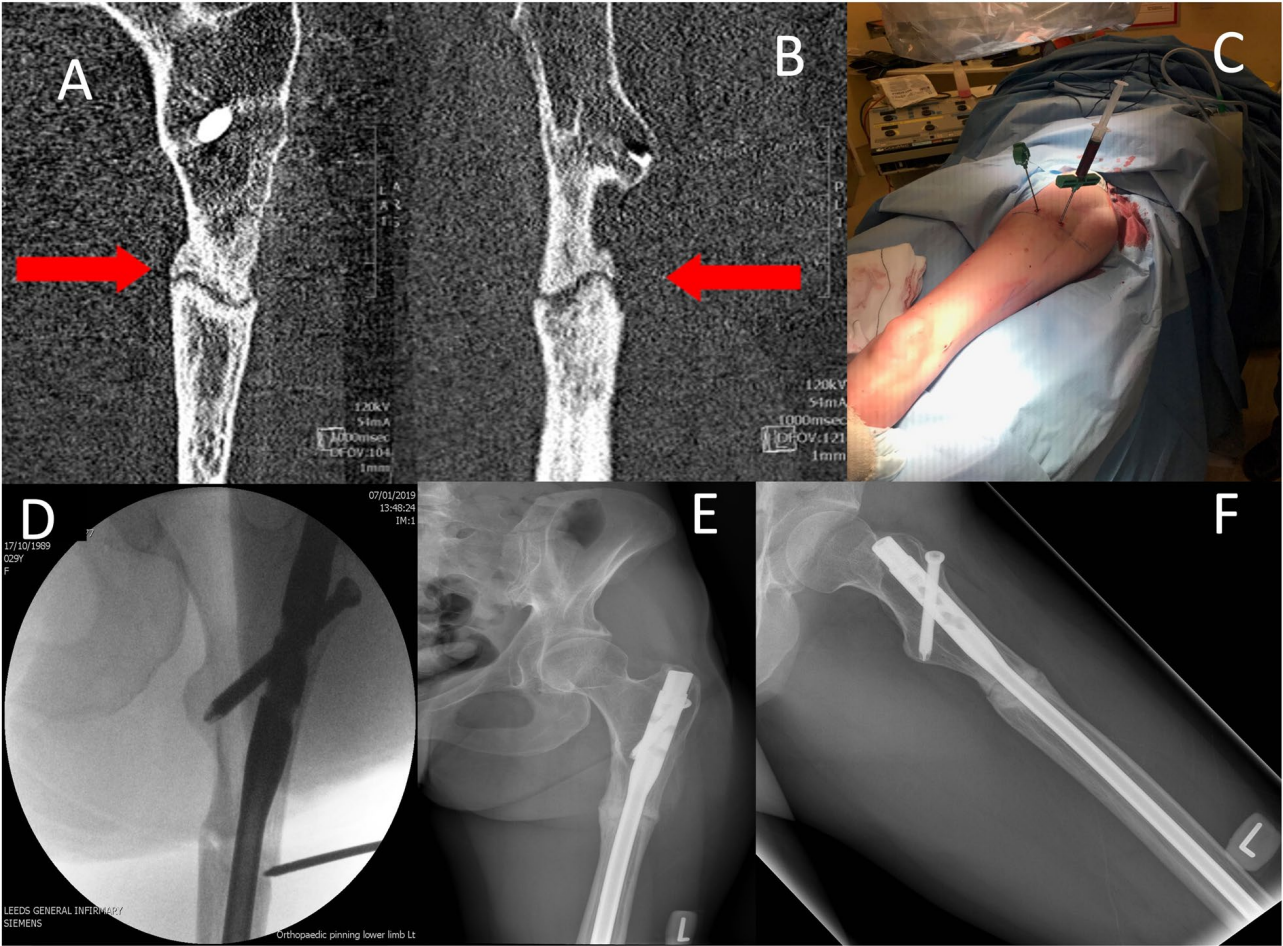
A 42-year-old male sustained a closed distal tibial fracture following a fall (**A**, **B**). The fracture was stabilised with a MIPO plate and demonstrated little callus formation 6 weeks after surgery (**C**, **D**). A CT scan taken at 6 months demonstrates an established nonunion (**E**). The patient underwent the harvesting of 60 ml bone marrow from the ipsilateral iliac crest, which was concentrated down to 7 ml of BMAC. This was injected by a percutaneous technique into the nonunion site (**F**, **G**). Following this technique, union was achieved within 4 months (**H**, **I**). *MIPO* Minimal invasive plate osteosynthesis

**Fig. 3:**
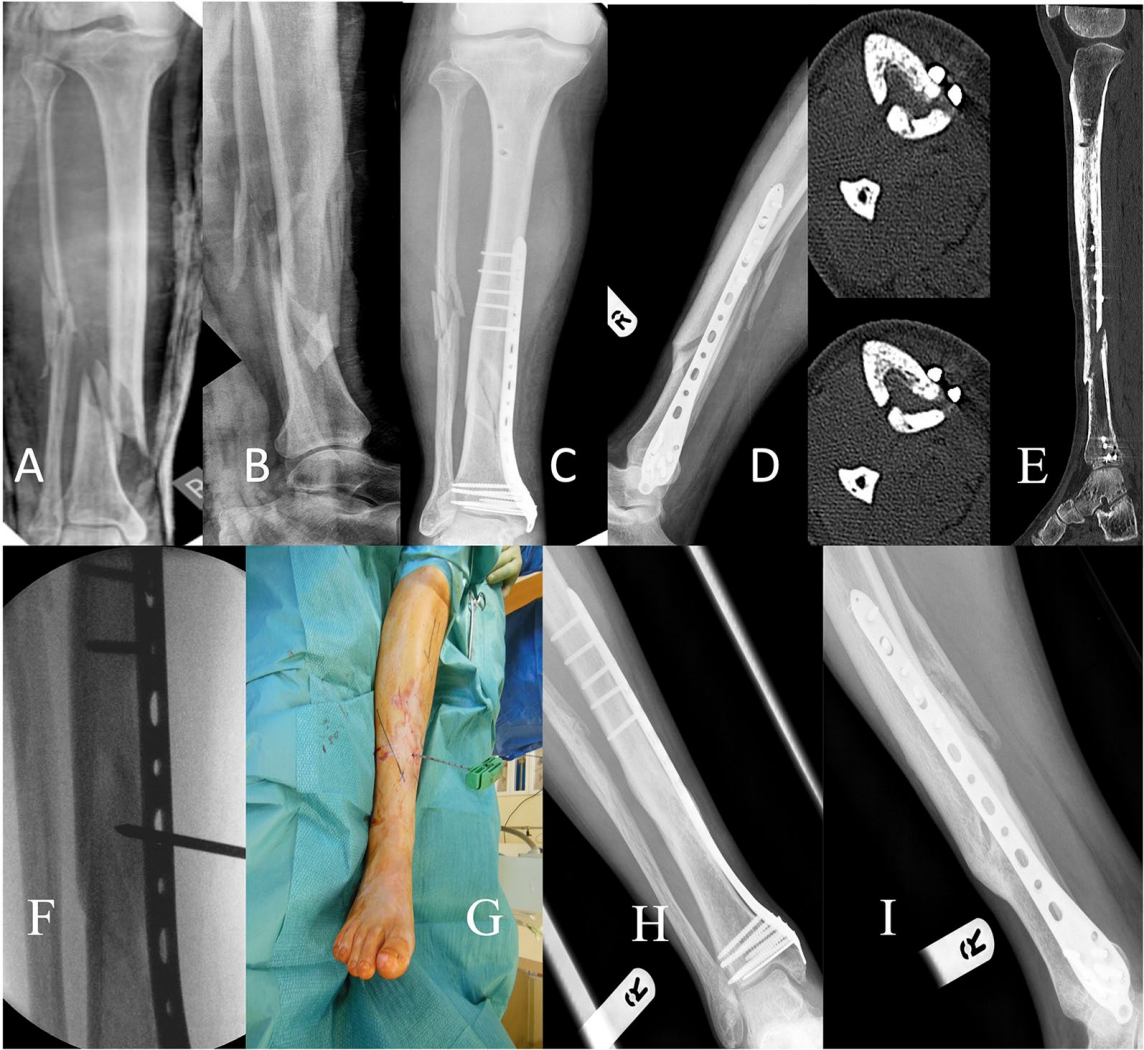
A 30-year-old female presented a CT-proven atrophic nonunion at 9 months following an IM nail for a closed femoral fracture (**A**, **B**). The patient underwent the harvesting of 60 ml of bone marrow, which was concentrated down to 8 ml volume and injected into the nonunion site (**C**, **D**). Images **E** and **F** demonstrate union at 3 months post-operation. *IM* Intramedullary

In its simplest form, as a source of MSCs, marrow aspirate can be injected directly into the nonunion site [34]. Using this technique, Singh et al. achieved union in 10/12 patients that they treated with percutaneous bone marrow aspirate delivery into long-bone nonunion [35]. Hau et al. also employed marrow aspirate laden onto freeze-dried allograft as a carrier in their cohort of long-bone nonunions (nine of the femur, six of the tibia, two of the ulna and one of the humerus) [36]. Even when compared to the gold standard of autologous bone graft, they saw accelerated bone healing with marrow aspirate, reducing the average time to union by 28%. Figure 2 demonstrates the percutaneous application of bone marrow aspirate to a tibial nonunion, whereas Fig. 3 shows the percutaneous application of bone marrow aspirate to a femoral nonunion.

Culture-expanded MSCs, whilst more resource intensive, are beneficial in the management of fracture nonunion. Emadedin et al. injected culture-expanded MSCs into three femoral and two tibial nonunions, achieving radiological union in 3/5 cases [37]. In their series of three patients with tibial/femoral nonunion, Wittig et al. achieved a 100% union rate within 12 months following the injection of culture-expanded MSCs seeded onto collagen microspheres as an osteoconductive scaffold [38]. Similarly, Giannotti et al. loaded culture-expanded MSCs onto fibrin clot scaffolds augmented with autologous or synthetic bone graft in the management of eight patients with forearm and humeral nonunion. They were able to achieve union in 8/9 patients without further intervention by adopting this strategy [39]. Ismail et al. compared culture-expanded MSCs loaded on hydroxyapatite granules with autologous bone graft in ten patients with long-bone nonunion [40]. Even against the current gold standard, union was achieved significantly faster when using MSCs on a scaffold, with greater functional improvements also achieved in the first 4 months post-surgery. Finally, Gomez-Barrena et al. seeded culture-expanded MSCs on 20% hydroxyapatide/80% β-TCP scaffolds in 28 patients with long-bone nonunion [41]. They achieved union in 26/28 patients at 12 months, with excellent clinical outcomes.

With concerns about potential donor-site morbidity, the use of allogenic MSCs remains an option, though they also have associated concerns regarding disease transmission and immunogenicity. To investigate the role of allogenic MSCs, Jayankura et al. percutaneously injected allogenic MSCs (ALLOB, Bone Therapeutics) into 22 patients with long-bone nonunion [42]. Using this technique, they achieved union in 20/22 patients at 6 months, although they did note increased in anti-human leukocyte antigen antibodies in 23% of patients, albeit with no clinical hypersensitivity reactions. At present, the literature is limited with regards to allogenic MSCs, and therefore they should be used with caution, particularly when repeated doses are being considered [43].

### MSCs in bone defects

As with nonunion, bone defects are difficult to manage, as they require osteogenic cells, osteoinductive mediators and, importantly, an appropriate osteoconductive scaffold to bridge the existing gap until union is achieved. Current techniques for managing bone defects include bone transport, which takes advantage of the tension-stress principle (whereby continuous tensile stress results in callus as two bone segments are gradually moved away from one another) or bone grafting, be that acute or via a two-stage induced-membrane technique. Both of these techniques, whilst effective, carry high morbidity, both during and following treatment. As such, there is still interest in generating additional osteogenic stimulus in the management of bone defects to accelerate bone healing (Tables 5 and 6).

**Table 5:** Papers assessing the use of cell therapy in the management of bone defects

Authors	Procedure	Sites treated	Size of defect	Source of MSCs	Supplementary therapy
Shabaan et al. 2023 [44]	Iliac crest bone graft ± BMAC for alveolar cleft defects	Alveolar defects (18 in experimental group, 18 in control)	Not reported	Posterior iliac crest; direct injection	Cells combined with autologous iliac crest graft; control group had autologous iliac crest graft only
Utomo et al. 2019 [49]	Marrow aspirate + freeze-dried allograft for traumatic long-bone defects	Tibia—1 Humerus—1	Tibia—6cm Humerus—5cm	Anterior iliac crest; direct combination with allograft	IM nail for tibia, plate fixation for humerus. Cells combined with freeze-dried allograft + platelet-rich plasma
Sponer et al. 2018 [46]	Scaffold laden with MSCs vs allograft for proximal femoral bone defect reconstruction during revision arthroplasty	All proximal femur Experimental group—19 Control—9	Experimental—14.4 cm^3^ Control—29 cm^3^	Anterior iliac crest; culture expanded until passage 4 and then combined with β-TCP scaffold	Scaffolds also soaked in autologous blood Control group had allograft only
Bajestan et al. 2017 [45]	Autogenous bone graft vs MSCs + synthetic scaffold for alveolar cleft defects	Alveolar defects (10 in experimental group, 8 in control)	Experimental—2.9 mm Control—3.6 mm	Posterior iliac crest; expanded in culture for 12 days, combined with β-TCP scaffold	Nil Control group had autogenous bone block grafting only
Dufrane et al. 2015 [47]	MSC + demineralised bone matrix for post-tumour bone/pseudoarthrosis defects	Tibia—4 Femur—1 Ulna—1	Not reported	Adipose-derived MSCs; expanded in culture until passage 4, combined with DBM scaffold	Bone tumour patients underwent intercalary replacement; MSC/DBM mix was added to peripheries to aid integration; all tumour patients also had allograft
Marcacci et al. 2007 [48]	MSC + hydroxyapatite scaffold for long-bone defects	Ulna—2 Humerus—1 Tibia—1	5.25 cm	Anterior iliac crest aspiration; culture expanded and combined with hydroxyapatite scaffold	External fixation in all cases; no other supplementary therapy

*IM* Intramedullary

**Table 6:** Outcomes following the use of cell therapies for the management of bone defects

Authors	Duration of follow-up	Radiological outcome	Clinical outcome
Shabaan et al. 2023 [44]	12 months	Significantly higher bone volume + bone density in the experimental group	No complications in either group
Utomo et al. 2019 [49]	8 months	Radiological union achieved in both cases (timeline not described)	Both patients regained normal function without ongoing pain
Sponer et al. 2018 [46]	21 months	No difference in graft incorporation at 1 year	No difference in clinical or patient-reported outcomes (Harris hip score)
Bajestan et al. 2017 [45]	4 months	Not reported	Gain of horizontal augmentation was greater in the control group (3.3 mm) compared to the experimental group (1.5 mm) Augmentation allowed the placement of implants in all control cases, but in just 5/10 of cases in the experimental group
Dufrane et al. 2015 [47]	37 months	Incorporation of all tumour prosthesis Failure of union in 2/3 pseudoarthrosis cases	1/3 with intercalary replacement developed a deep infection requiring removal of the implant 2/3 with pseudoarthrosis developed a nonunion requiring revision
Marcacci et al. 2007 [48]	60 months	Consolidation achieved in all cases between 5 and 7 months (average 6 months)	No complications reported

Dealing with smaller alveolar cleft defects within reconstructive dentistry, both Shabaan et al. and Bajestan et al. employed cell therapies to try and reduce a defect size prior to siting implants [44, 45]. Shabaan utilised marrow aspiration, whilst Bajestan utilised culture-expanded MSCs on β-TCP scaffolds. Shabaan demonstrated significantly higher bone density with the use of MSCs when compared to bone graft alone; however, Bajestan noted that the use of MSCs with a synthetic bone substitute resulted in inadequate defect reconstruction compared to bone graft and was unable to site an implant in half of the cases.

Sponer et al. utilised culture expanded MSCs on β-TCP scaffolds and compared this to allograft in the management of proximal femoral bone defects following revision arthroplasty [46]. In the 19 patients managed with cell therapy, they noted similar graft incorporation at 1 year and no difference in the Harris hip score, suggesting that cell therapy could be safely used as an alternative to allograft, avoiding the risks of contamination or immunogenicity. Dufrane et al. utilised culture-expanded MSCs combined with DBM to facilitate the incorporation of intercalary replacement following bone tumour resection and resection of pseudoarthrosis, and they demonstrated excellent incorporation around the tumour prosthesis but poor outcomes with a high rate of failure (2/3) when utilised to reconstruct defects in pseudoarthrosis [47].

In the field of trauma, Marcacci et al. utilised culture-expanded MSCs combined with hydroxyapatite scaffolds to manage long-bone defects in four patients (average defect size: 5.25 cm; two ulna, one tibia, one humerus) [48]. All four cases achieved consolidation within 7 months of the procedure, with no reported complications. Similarly, Utomo et al. reconstructed one tibial (6 cm) and one humeral (5 cm) defect combining bone marrow aspirate with freeze-dried allograft and platelet-rich plasma, achieving union with good functional results in both cases [49]. The literature surrounding cell therapies in the management of bone defects is limited, and they are often used in conjunction with other techniques. The addition of an osteogenic stimulus does appear to provide a benefit, particularly in cases where this is combined with bone graft, although further larger series are required to demonstrate this.

### MSCs in avascular necrosis of the femoral head

Avascular necrosis (AVN) of the femoral head occurs as a result of disruption to the blood supply, which leads to osteocyte death. It may occur spontaneously, following trauma, or due to risk factors such as corticosteroid use or alcoholism [50]. Early AVN is often an incidental finding on a magnetic resonance imaging (MRI) scan, with the disease progressing to femoral head sclerosis, subchondral fracture and, eventually, femoral head collapse. In its early stages, management is based on joint-preserving procedures, including core decompression, vascularised graft or re-directional osteotomies. Once femoral head collapse occurs, joint-preserving procedures become ineffective, and arthroplasty becomes the treatment option of choice [50]. In the setting of AVN, cell therapies provide an exciting avenue to regenerate subchondral bone, preventing femoral head collapse and therefore the requirement for future arthroplasty (Tables 7 and 8).

**Table 7:** Papers assessing the use of cell therapy in the management of avascular necrosis of the femoral head

Authors	Procedure	Classification	Source of MSCs	Supplementary therapy
Li et al. 2021 [59]	Experimental: core decompression, angioconductive bioceramic rod + bone marrow buffy coat Control: core decompression, angioconductive bioceramic rod + β-TCP granules	Experimental: Ficat 1: 1 Ficat 2: 19 Ficat 3: 2 Control: Ficat 1: 1 Ficat 2: 20 Ficat 3: 6 Ficat 4: 2	Anterior iliac crest; injected directly	Experimental group also received iliac crest bone graft mixed with the marrow aspirate
Hauzer et al. 2020 [58]	Experimental: core decompression + culture-expanded osteoblastic cells Control: core decompression + BMAC	Experimental: ARCO 1: 10 ARCO 2: 17 Control: ARCO 1: 10 ARCO 2: 16	Anterior or posterior iliac crest; BMAC directly injected, osteoblastic cell culture expanded	None
Li et al. 2020 [61]	Experimental: core decompression, bone graft + bone marrow buffy coat Control: core decompression + bone graft	Experimental: Ficat 2: 11 Ficat 3: 10 Control: Ficat 2: 11 Ficat 3: 9	Anterior iliac crest; injected directly	None
Hauzeur et al. 2018 [56]	Experimental: core decompression + BMAC Control: core decompression + saline	All ARCO stage 3 (23 total)	Anterior iliac crest; injected directly	None
Chen at el 2016 [51]	Umbilical cord MSCs injected into femoral artery	ARCO 2: 5 ARCO 3A: 4	Umbilical-cord-derived culture-expanded MSCs	None
Gao et al. 2016 [57]	Core decompression, bone marrow aspirate + rh-BMP-2	ARCO 1: 3 ARCO 2: 21 ARCO 3: 27	Not stated	rhBMP-2
Mao et al. 2015 [53]	Experimental: porous tantalum rod + infusion of peripheral blood stem cells into medial circumflex artery Control: porous tantalum rod only	Experimental: ARCO 1: 8 ARCO 2: 29 ARCO 3A: 11 Control: ARCO 1: 10 ARCO 2: 23 ARCO 3A: 8	Peripheral blood stem cells mobilised by granulocyte colony-stimulating factor	None
Zhao et al. 2015 [62]	Porous tantalum rod with MSCs + vascularised iliac graft	ARCO 3C: 19 ARCO 4: 12	Posterior iliac crest; culture expanded	Vascularised iliac crest graft
Daltro et al. 2015 [54]	Bone marrow injection only	Ficat 0: 20 Ficat 1: 31 Ficat 2A: 16 Ficat 2B: 22	Posterior iliac crest; injected directly	None
Li et al. 2015 [55]	Bone marrow + adipose-derived stem cells + PRP	Ficat 1: 5 Ficat 2: 4 Ficat 3: 3 Ficat 4: 3	Anterior iliac crest + adipose; injected directly	PRP
Aoyama et al. 2014 [60]	MSCs on β-TCP in combination with vascularised iliac crest bone graft	ARCO 3a: 5 ARCO 3b: 4	Posterior iliac crest; culture expanded + seeded on β-TCP	Vascularised iliac crest graft
Mao et al. 2013 [52]	Bone marrow mononuclear cells injected via the medial circumflex artery	Ficat 1: 16 Ficat 2: 52 Ficat 3: 10	Anterior iliac crest; injected directly	None

**Table 8:** Outcomes following the use of cell therapies for the management of avascular necrosis of the femoral head

Authors	Duration of follow-up	Radiological outcome	Clinical outcome	Conversion to THR
Li et al. 2021 [59]	5 years	Not recorded	Experimental group had significantly higher Harris hip scores at final follow-up (84 vs 73)	Experimental—4.5% Control—17.2%
Hauzer et al. 2020 [58]	36 months	Progression to ARCO 3 or beyond: Experimental—22% Control—46%	No difference in clinical outcomes between the two groups with regards to VAS score or WOMAC score	Experimental—15% Control—35%
Li et al. 2020 [61]	10 years	Radiological progression: Experimental—24% Control—50%	VAS score was significantly lower in the experimental group at all post-operative time points Functional outcomes were significantly better in the experimental group	Experimental—9.5% Control—20%
Hauzeur et al. 2018 [56]	24 months	Radiological progression to stage 4: Experimental—43% Control—43%	No difference in WOMAC score between the experimental and control groups	15/23 progressed to needing THR No difference between groups
Chen et a.l 2016 [51]	24 months	Necrotic area reduced on MRI at both 12 and 24 months	Oxygen delivery increased within 3 days of injection	Na
Gao et al. 2016 [57]	6.8 years	17.6% of hips demonstrated progressive collapse of the femoral head	Clinical success rate was 95.8% for ARCO stages 1 and 2, 83.3% for stage 3A, and 66.7% for stage 3B	1 patient required THR
Mao et al. 2015 [53]	36 months	Radiological progression: Experimental—4/48 Control—13/41	Harris hip score was significantly higher in the experimental group	Experimental—6.25% Control—21.95%
Zhao et al. 2015 [62]	5 years	Radiological progression in 3/19 stage 3C hips and 5/12 stage 4 hips	Harris hip score improved from 39 pre-op to 77 post-op	5/31 at 5 years
Daltro et al. 2015 [54]	5 years	No patient experienced disease progression on XR or MRI	Significant improvement in Harris hip score was maintained to 60 months	0 conversion to THR
Li et al. 2015 [55]	12 months	No radiological progression	Not reported	0 conversion to THR
Aoyama et al. 2014 [60]	24 months	Stable size of lesion at 1 and 2 years	JOA score improved from 66 at baseline to 90 at 1 year and 88 at 2 years	0 conversion to THR
Mao et al. 2013 [52]	5 years	Radiological progression: Stages 1 + 2: 4% Stage 3: 30%	Mean HHS: Baseline—59 12 months—75 24 months—82 36 months—81 48 months—79 60 months—74	6/78 (7.7%)

*WOMAC* Western Ontario and McMaster Universities Osteoarthritis Index, *THR* Total hip arthroplasty, *JOA* Japanese Orthopaedic Association, *Na* Not available

MSCs can be delivered to the femoral head via several mechanisms, including injection into the local circulation, percutaneous injection into the offending lesion, or application on scaffolds following core decompression. Three recent studies have examined the use of local injection of MSCs for femoral head AVN. Chen et al. injected umbilical cord MSCs into the femoral artery in nine patients with stage 2 and stage 3 AVN [51]. In all patients, the AVN lesion remained stable over 24 months of follow-up, with no patient requiring conversion to total hip arthroplasty (THA). Mao et al. similarly injected bone-marrow-derived mononuclear cells into the medial circumflex artery in 78 patients with grade 1–3 AVN, and they demonstrated that there was little progression in stage 1 and 2 hips at 5 years and a requirement for THA in just 7.7% across all stages [52]. Two years later, the same group also published their results on porous tantalum rod insertion ± infusion of peripheral blood stem cells into the medial circumflex femoral artery, demonstrating that there was an improved radiological and clinical outcome in the infusion group at 36 months [53]. Within this cohort, the requirement for THA was reduced from 22% in the control group to 6% in the infusion group. A number of authors have also examined the injection of cells only into the necrotic lesion. Dalto et al. injected minimally manipulated bone marrow into the hips of 89 patients with stage 1 and 2 disease; they demonstrated that lesions were stable in all patients at 5 years and that there were no conversions to THA [54]. Li et al. obtained similar results at a year following the injection of bone marrow, adipose-derived stem cells and PRP [55].

Decompression is a key feature of the early management of AVN. A number of authors have applied marrow or expanded MSCs following decompression to try and stimulate healing in the subchondral area. Hauzer et al. compared core decompression with bone marrow aspirate concentrate (BMAC) injection to core decompression and saline, demonstrating that there was no difference between the two groups with regards to radiological progression, clinical outcomes or need for THA [56]. It should be noted, however, that all patients in this cohort had stage 3 disease. Gao et al. similarly injected marrow aspirate supplemented with recombinant bone morphogenetic protein-2 (rhBMP-2) into adolescent patients following core decompression, achieving good clinical outcomes for 96% and 83% of stage 1 and stage 2 hips, respectively, at 6.8 years [57]. Outcomes were poorer for stage 3 hips, with only 67% achieving a good outcome; however, only one patient had proceeded to THA. Hauzer et al. also examined the use of expanded osteoblastic cells vs BMAC in patients undergoing core decompression for stage 1 and 2 AVN [58]. Outcomes were significantly better with culture-expanded cells, with fewer demonstrating radiological progression and just 15% requiring THA, compared to 35% in the BMAC group.

To further provide osteoconductivity, various authors have utilised both native and synthetic material as scaffolds to try and further improve the bone healing response. Li et al. combined bone marrow buffy coat with an angioconductive bioceramic rod and compared this to a control of β-TCP granules without marrow [59]. Within this cohort, patients managed with bone marrow had significantly higher Harris hip scores at 5 years (84 vs 73) and a significantly lower conversion rate to THA (4.5% vs 17.2%). Aoyama examined the use of culture-expanded MSCs seeded on β-TCP scaffolds combined with vascularised iliac crest graft in nine patients with stage 3 disease [60]. At 2 years, all patients remained functionally high-performing, with no radiological progression and no conversions to THA. Both Li et al. and Zhao et al. examined the combination of culture-expanded MSCs with bone graft [61, 62]. In a 10-year follow-up of core decompression, bone graft ± BMAC, Li established that the addition of marrow reduced radiological progression from 50 to 24%, produced significantly better functional outcomes, and reduced the requirement for arthroplasty from 20% to just 9.5%. Similarly, when treating exclusively stage 3C and 4 hips, Zhao demonstrated that a combination of a porous tantalum rod with culture-expanded MSCs and vascularised iliac crest graft prevented radiological progression in 8/31 hips, with a THA rate of just 16% at 5 years.

Cell therapy is particularly exciting in the management of early AVN, as it demonstrates strong utility in preventing progression beyond stage 1 and 2 disease. Nonetheless, when combined with other gold standard techniques such as autologous bone grafting, it can still provide value, even in advanced disease.

### Other applications of MSCs

Whilst cell therapies are currently most commonly utilised in the management of AVN and nonunion, they have wide-ranging potential (Tables 9 and 10). Both Di Bella et al. and Li et al. utilised bone marrow for the management of paediatric bone cysts [63, 64]. In both of these series, the addition of bone marrow resulted in improved healing compared to the current standard of management. Lee et al. also demonstrated great efficacy of BMAC during distraction osteogenesis [65]. Injection of BMAC at the time of osteotomy in this cohort of 20 patients undergoing bilateral tibial lengthening over a nail resulted in faster cortical consolidation and a faster return to full weight-bearing. Cell therapies have also been trialled in spinal fusion as an alternative to autologous graft. Unfortunately, however, despite positive clinical outcomes, the nonunion rate was 61%, and therefore their use is not routinely supported for this indication [66].

**Table 9:** Miscellaneous papers assessing the use of cell therapy

Authors	Procedure	Sites treated	Source of MSCs	Supplementary therapy
Di Bella et al. 2010 [63]	Corticosteroid vs autologous marrow + DBM for unicameral bone cysts	Experimental: Humerus—29 Femur—11 Other—1 Control: Humerus 108 Femur—31 Other—4	Anterior iliac crest; directly injected	MSCs combined with DBM for injection Control group had injection of corticosteroid only
Li et al. 2016 [64]	Autologous marrow vs titanium elastic nail for simple bone cysts	Experimental: Humerus—16 Femur—7 Control: Humerus—14 Femur—9	Anterior iliac crest; directly injected	No supplementary therapy Control group had a titanium elastic nail only
Lee et al. 2014 [65]	BMAC to improve regeneration in patients undergoing bilateral tibial lengthening over a nail	Tibia—40 (20 patients)	Anterior iliac crest; directly injected	Supplemented with platelet-rich plasma, lengthened at a rate of 1 mm/day Similar final lengthening
Thaler et al. 2013 [66]	Lumbar decompression + fusion with autologous marrow + β-TCP scaffold	Lumbar spine: Single level—26 Two levels—5 Three levels—3	Anterior iliac crest; directly injected	PEEK cage pre-filled with β-TCP

**Table 10:** Outcomes of miscellaneous papers assessing the use of cell therapy

Authors	Duration of follow-up	Radiological outcome	Clinical outcome
Di Bella et al. 2010 [63]	Experimental—20 months Control—48 months	Greater rate of healing in experimental group (59% vs 21%)	Fewer treatment failures (defined as refracture, no evidence of healing at 6 months, or recurrence of the cyst that requires additional treatment) in experimental group
Li et al. 2016 [64]	Not reported	Experimental: Complete healing—61% Partial healing with small residual—26% Control: Complete healing—70% Partial healing with small residual—17%	In both cohorts, there were three recurrences requiring further treatment (all had autologous bone marrow injection) No difference in overall complication rate
Lee et al. 2014 [65]	24 months	Similar external fixator index for the two groups Faster cortical consolidation in the experimental group (0.95 vs 1.34 months/cm)	Experimental group returned to full weight-bearing significantly more quickly (full weight-bearing index 0.99 months/cm in the experimental group vs 1.38 months/cm in the control group)
Thaler et al. 2013 [66]	12 months	61% fusion rate at 12 months on CT	Oswestry disability index: Baseline—62 3 months—24 12 months—14 VAS back pain: Baseline—8/10 12 months—3/10 VAS leg pain: Baseline 6/10 12 months—2/10

## Conclusion

Cell therapy continues to be an exciting avenue for augmenting bone repair, spanning several key indications. At present, the literature remains heterogeneous, with the majority of publications in this area being based on small series, with a high risk of bias. Ex-vivo expansion of cells onto custom scaffolds is a desirable end point for the future of this technology; however, the processes to facilitate this are laborious and costly. The combination of ex-vivo expanded cells with autologous bone provides a clinically effective alternative, though at the cost of donor-site morbidity. Moving forward, research should seek to answer key questions, including how we can better purify the marrow aspirates without the need for tissue culture, and seek to identify improved biocompatible scaffolds that perform similarly to native bone.

## Data Availability

Not applicable.

## Abbreviations

MSC: Mesenchymal stromal cell
DBM: Demineralised bone matrix
PRP: Platelet-rich plasma
ECM: Extracellular matrix
β-TCP: β-Tricalcium phosphate
PLA: Polylactic acid
PCL: Polycaprolactone
CAD: Computer-aided design
PTH: Parathyroid hormone
AVN: Avascular necrosis
DASH: Disabilities of the arm, shoulder and hand
FDA: Food and Drug Administration
MRI: Magnetic resonance imaging
THA: Total hip arthroplasty
BMAC: Bone marrow aspirate concentrate
rhBMP-2: Recombinant bone morphogenetic protein-2
